# *Arabidopsis thaliana PGR7* Encodes a Conserved Chloroplast Protein That Is Necessary for Efficient Photosynthetic Electron Transport

**DOI:** 10.1371/journal.pone.0011688

**Published:** 2010-07-21

**Authors:** Hou-Sung Jung, Yuki Okegawa, Patrick M. Shih, Elizabeth Kellogg, Salah E. Abdel-Ghany, Marinus Pilon, Kimmen Sjölander, Toshiharu Shikanai, Krishna K. Niyogi

**Affiliations:** 1 Department of Plant and Microbial Biology, University of California, Berkeley, California, United States of America; 2 Graduate School of Agriculture, Kyushu University, Fukuoka, Japan; 3 Department of Botany, Graduate School of Science, Kyoto University, Kyoto, Japan; 4 Department of Bioengineering, University of California, Berkeley, California, United States of America; 5 Biology Department, Colorado State University, Fort Collins, Colorado, United States of America; 6 Physical Biosciences Division, Lawrence Berkeley National Laboratory, Berkeley, California, United States of America; Umeå Plant Science Centre, Sweden

## Abstract

A significant fraction of a plant's nuclear genome encodes chloroplast-targeted proteins, many of which are devoted to the assembly and function of the photosynthetic apparatus. Using digital video imaging of chlorophyll fluorescence, we isolated *proton gradient regulation 7* (*pgr7*) as an *Arabidopsis thaliana* mutant with low nonphotochemical quenching of chlorophyll fluorescence (NPQ). In *pgr7*, the xanthophyll cycle and the *PSBS* gene product, previously identified NPQ factors, were still functional, but the efficiency of photosynthetic electron transport was lower than in the wild type. The *pgr7* mutant was also smaller in size and had lower chlorophyll content than the wild type in optimal growth conditions. Positional cloning located the *pgr7* mutation in the *At3g21200* (*PGR7*) gene, which was predicted to encode a chloroplast protein of unknown function. Chloroplast targeting of PGR7 was confirmed by transient expression of a GFP fusion protein and by stable expression and subcellular localization of an epitope-tagged version of PGR7. Bioinformatic analyses revealed that the PGR7 protein has two domains that are conserved in plants, algae, and bacteria, and the N-terminal domain is predicted to bind a cofactor such as FMN. Thus, we identified *PGR7* as a novel, conserved nuclear gene that is necessary for efficient photosynthetic electron transport in chloroplasts of Arabidopsis.

## Introduction

The photosynthetic apparatus of plants consists of several large multiprotein thylakoid membrane complexes that are composed of proteins encoded by both the nuclear and chloroplast genomes. Because chloroplasts in plants are derived from the endosymbiosis of a cyanobacterium by a eukaryotic heterotroph more than 1 billion years ago [1], most of the structural components of the photosynthetic complexes were originally encoded by the genome of the cyanobacterial endosymbiont. Through a process of endosymbiotic gene transfer, the vast majority of photosynthesis genes were transferred to the host nucleus [2], where they acquired sequences encoding N-terminal chloroplast transit peptides that allowed for posttranslational import of the proteins into the organelle. The chloroplast genomes of plants have retained fewer than 100 protein-coding genes, many of which are necessary for expression of an even smaller subset of chloroplast-encoded photosynthesis genes. A number of nuclear genes are also involved in expression of the chloroplast genome at multiple levels, including transcription, mRNA maturation, translation, targeting, and assembly of complexes that comprise a functional photosynthetic electron transport system.

Electron transport in chloroplasts is necessary not only for NADPH and ATP production in photosynthesis, but also for photoprotection. Photosynthetic electron transport in excess light generates a high pH gradient across the thylakoid membrane that is critical for the thermal dissipation of excess absorbed light energy [3]. The resulting low pH in the thylakoid lumen activates violaxanthin de-epoxidase (VDE), and the activated VDE synthesizes zeaxanthin (Z) and antheraxanthin (A) from violaxanthin (V) in the xanthophyll cycle [4]. The low pH also drives proton binding to photosystem II (PSII) components including the PsbS protein [5], [6]. Both protonation and Z binding cause conformational changes that are involved in the thermal dissipation, which can be quantified by measuring nonphotochemical quenching of chlorophyll fluorescence (NPQ) [3].

The isolation and characterization of *Arabidopsis thaliana* mutants that are defective in NPQ has proven to be a useful approach for the elucidation of factors that are involved in NPQ, including various electron transport components that affect ΔpH generation. For example, characterization of the *proton gradient regulation 1* (*pgr1*) mutant conditionally defective in the function of cytochrome *b_6_f* complex showed that the full activity of the complex is important for generating the necessary ΔpH for NPQ [7]. A chloroplast copper transporter was identified by a complementation group of NPQ-deficient mutants, which affect electron transport by restricting the supply of copper for holoplastocyanin assembly in the thylakoid lumen [8]. The involvement of photosystem I (PSI) cyclic electron transport in NPQ has also been shown with the *pgr5* mutant, which is defective in one of two pathways of cyclic electron transport around PSI [9].

In this study, a novel factor involved in photosynthetic electron transport has been identified using Arabidopsis molecular genetics. The *pgr7* mutant was initially isolated as a low NPQ mutant using video imaging of chlorophyll fluorescence. Positional cloning led to the identification of the affected gene in the mutant. The *PGR7* gene encodes an “unknown protein” conserved in plants. PGR7 contains a transit peptide for chloroplast localization and a predicted FMN-binding domain. Physiological and biochemical experiments with the *pgr7* mutant showed that *PGR7* is necessary for efficient photosynthetic electron transport.

## Results

### Phenotypes of the *pgr7* mutant

**Figure 1:**
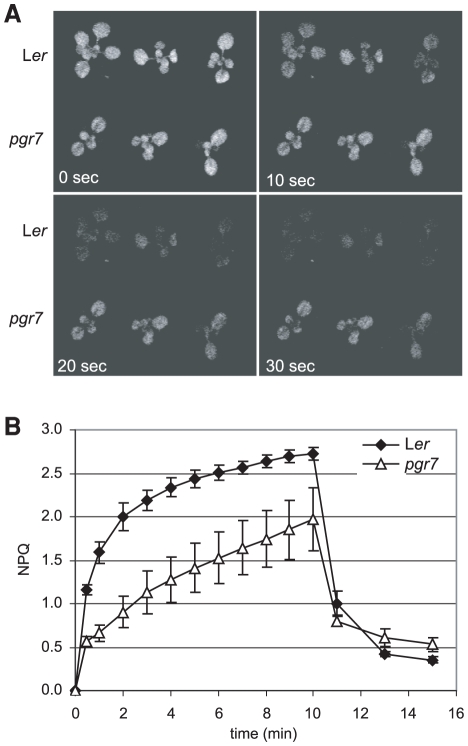
Low nonphotochemical quenching of chlorophyll fluorescence (NPQ) phenotype of the *pgr7* mutant. A) Digital video images of chlorophyll fluorescence quenching in *pgr7* and wild type, L*er*. Seedlings on MS agar plates were dark-adapted for 30 min and then were illuminated with actinic light (500 µmol photons m^−2^ s^−1^). Chlorophyll fluorescence images were captured by using a CCD camera with a far-red transmitting filter [13]. Images captured at the onset of illumination (0 sec) and at 10, 20 and 30 sec are shown. B) NPQ values in *pgr7* and L*er*. NPQ values were measured using an FMS2 fluorometer during actinic illumination with 1200 µmol photons m^−2^ s^−1^ for 10 min, followed by relaxation in the dark for 5 min. Each data point represents the mean ± SD (n = 4).

The *pgr7* mutant was isolated as a low NPQ mutant from fast neutron-mutated M_2_ plants by digital video imaging of chlorophyll fluorescence. Under actinic illumination, the chlorophyll fluorescence was quickly quenched in the wild type (L*er*), whereas the *pgr7* mutant emitted higher levels of chlorophyll fluorescence throughout the 30 sec illumination (Figure 1A). Further chlorophyll fluorescence measurements showed that *pgr7* exhibited much less NPQ than the wild type during a 10 min actinic illumination (Figure 1B).

**Figure 2:**
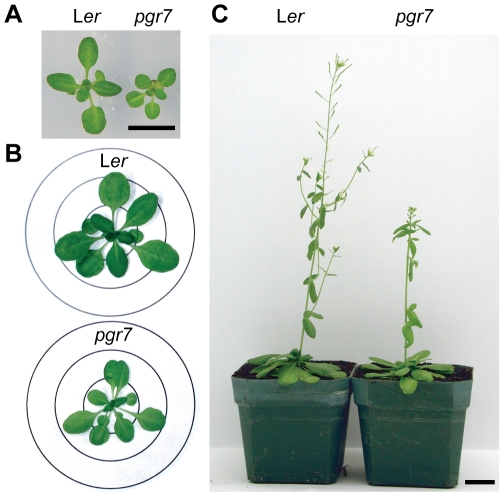
Comparisons of plant size and development between *pgr7* and L*er*. A) Seedlings grown on MS-salt agar plate for 2 weeks under continuous light. The bar represents 1 cm. B) Plants grown in soil in a short day condition (10 hours light/14 hours dark) for 4 weeks. The diameters of the circles are 2, 4 and 6 cm. C) Plants grown in soil in a short day condition (10 hours light/14 hours dark) for 8 weeks. The bar represents 2.5 cm.

Besides the low NPQ, the *pgr7* mutant is smaller than the wild type (L*er*). When *pgr7* was grown photoautotrophically on minimal agar medium, the mutant seedlings were smaller than L*er*, although the color of the leaves was indistinguishable between the mutant and the wild type (Figure 2A). Similarly, there were differences in plant size between *pgr7* and L*er* grown directly on soil in a short-day condition for 4 weeks (Figure 2B) or 8 weeks (Figure 2C) and in a long-day condition (data not shown). In addition, soil-grown *pgr7* plants were slightly pale green compared to the wild type. As expected, the chlorophyll content in *pgr7* was significantly (P<0.01) lower than that of wild type, however there was no significant difference in chlorophyll *a* to *b* ratio (Table 1).

**Table 1:** Photosynthesis-related Phenotypes of *pgr7*.

	F_v_/F_m_	Chl *a*+*b* * (µmol cm^−2^)	Chl *a*/*b* (mol mol^−1^)	V+A+Z (µmol cm^−2^)
L*er*	0.829±0.004	56.1±3.5	2.81±0.05	3.23±0.46
*pgr7*	0.831±0.002	47.7±2.7	2.88±0.06	3.37±0.28

*: P<0.01; mean ± SD (n = 4).

**Figure 3:**
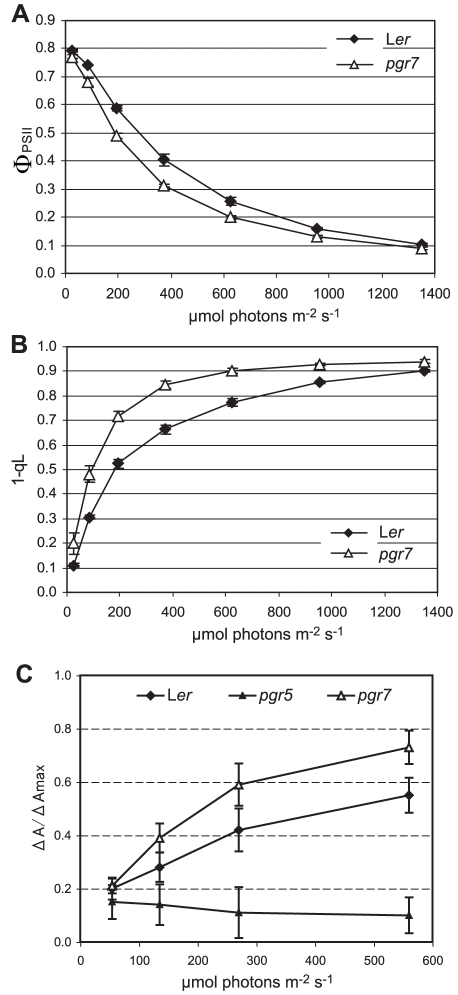
Light responses of Φ_PSII_, 1-qL and ΔA/ΔAmax in the *pgr7* mutant. Chlorophyll fluorescence parameters and P700^+^ absorbance were measured from attached rosette leaves during a 5 min illumination with each of the corresponding light intensities. Each data point is the mean ± SD (n = 3 except for n = 6 of *pgr7* in C). A) Quantum yield of PSII (Φ_PSII_) of *pgr7* and L*er*. B) Reduction state of PSII (1-qL) in *pgr7* and L*er*. C) Redox state of the reaction center chlorophylls of PSI (P700). ΔA/ΔAmax was determined by absorbance changes at 820 nm.

To understand the basis for the low NPQ in the *pgr7* mutant, we sought to determine whether any previously identified NPQ factors were affected in the *pgr7* mutant by comparing *pgr7* and L*er* plants grown on soil. To assess whether photosynthetic electron transport is affected in the *pgr7* mutant, we measured two key chlorophyll fluorescence parameters, the quantum yield of PSII (Φ_PSII_) and the reduction state of PSII (1-qL), as a function of light intensity (Figure 3). Although both *pgr7* and L*er* had an optimal value of the maximum quantum yield of PSII (F_v_/F_m_) (Table 1) [10], the Φ_PSII_ values in *pgr7* were significantly (P<0.05) lower than in wild type in the light (Figure 3A), reflecting a reduced rate of electron transport through PSII in *pgr7*. By measuring 1-qL, we also found significant changes in the redox state of the first stable electron acceptor of PSII, Q_A_, which reflects the redox state of the plastoquinone pool. The 1-qL in *pgr7* was much higher than that of the wild type at all light intensities, even at the growth light intensity (150 µmol photons m^−2^ s^−1^) (Figure 3B). These results indicate that electron transport is restricted in *pgr7* at some point after Q_A_ in PSII.

To clarify the defective point in the electron transport, light-intensity dependence of P700 oxidation was determined in *pgr7* and was compared with that in the wild type and *pgr5* (Figure 3C). The *pgr5* mutant is defective in PSI cyclic electron transport, which is essential for the induction of NPQ via its role in generating ΔpH [9]. In the wild type, P700 is completely reduced in the dark and becomes more oxidized during a series of increasing light intensities. The defect in PSI cyclic electron transport caused the reduction of P700 at higher light intensities in *pgr5*. In contrast, P700 in the *pgr7* mutant was more oxidized relative to the wild type at higher light intensities (Figure 3C). This result suggests that photosynthetic electron transport in *pgr7* is restricted between Q_A_ of PSII and P700 of PSI.

**Figure 4:**
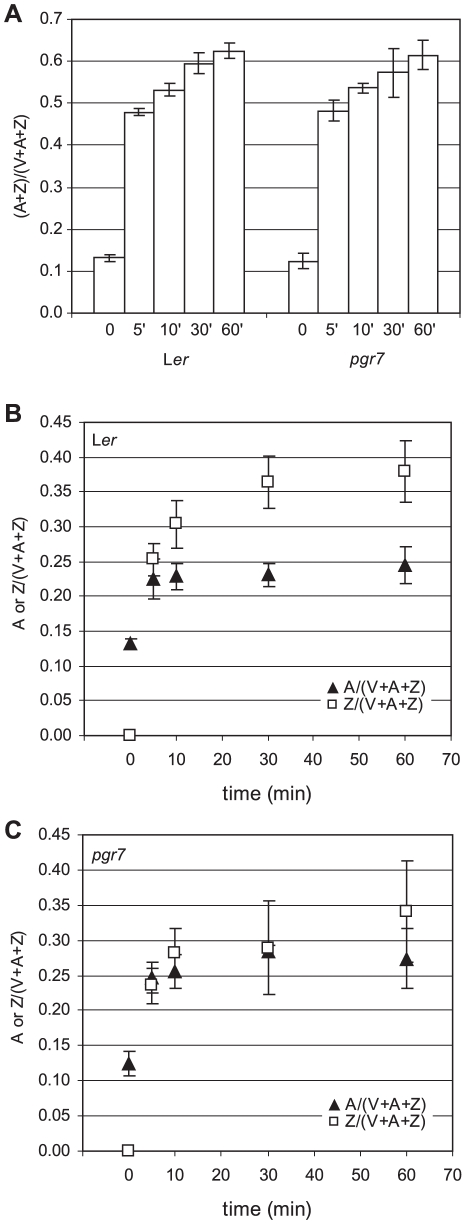
Characterization of the xanthophyll cycle in *pgr7*. The composition of the xanthophyll cycle pigments (Violaxanthin: V; Antheraxanthin; A; Zeaxanthin: Z) was determined in low light (LL: 150 µmol photons m^−2^ s^−1^) (0) and after being treated in high light (HL: 1100 µmol photons m^−2^ s^−1^) for 5 min (5′), 10 min (10′), 30 min (30′) and 60 min (60′). Each data point represents the mean of three measurements with SD. A) De-epoxidation state [(A+Z)/(V+A+Z)] of *pgr7* and L*er*. B) A (closed triangles) and Z (open squares) levels in L*er*. C) A (closed triangles) and Z (open squares) levels in *pgr7*.

A lower rate of electron transport might be expected to affect the generation of a high ΔpH and activation of the xanthophyll cycle enzyme, VDE, in the thylakoid lumen. The pH-dependent conversion of V to A and Z by VDE is necessary for full induction of NPQ [11], [12], [13], and the de-epoxidation state [(A+Z)/(V+A+Z)] is strongly correlated with NPQ [14]. To determine the de-epoxidation state, leaf disks of *pgr7* and L*er* were exposed to high light (1100 µmol photons m^−2^ s^−1^) for 5, 10, 30 and 60 min, and the amounts of V, A, and Z were determined using HPLC. Both *pgr7* and L*er* contained similar amounts of xanthophyll cycle pigments (V+A+Z) per leaf area (Table 1). During exposure to high light, there were no differences in the overall de-epoxidation state between *pgr7* and L*er* (Figure 4A), indicating that *pgr7* contains a functional xanthophyll cycle. However, there were differences in the relative levels of Z and A in *pgr7* and L*er*. In L*er*, Z/(V+A+Z) was much higher than A/(V+A+Z) at 10 min or more of high light treatment (Figure 4B). In contrast, in *pgr7*, Z/(V+A+Z) and A/(V+A+Z) were almost identical at 30 min (Figure 4C), and at 60 min, Z/(V+A+Z) was slightly higher than A/(V+A+Z) in *pgr7*, although the difference was smaller than that in L*er*. Thus, the composition of the de-epoxidated xanthophyll cycle pigments was different between *pgr7* and L*er* under high light, although there was no difference in the overall de-epoxidation state [(A+Z)/(V+A+Z)] (Figure 4).

**Figure 5:**
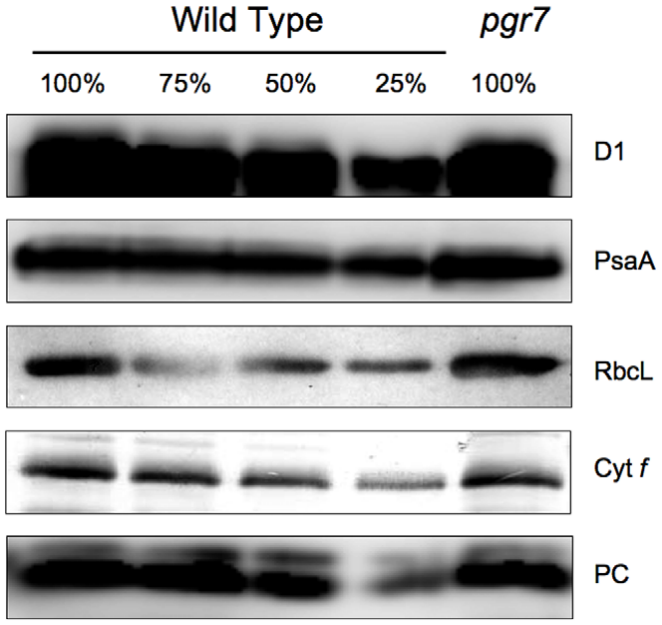
Immunoblot analysis of photosynthetic proteins. Photosynthetic proteins were detected using specific antibodies against D1 (PSII), PsaA (PSI), Cyt *f* (Cytochrome *b*_6_ *f* complex), RbcL (the large subunit of ribulose-1,5-bisphosphate carboxylase/oxygenase), and plastocyanin (PC). The lanes were loaded with chloroplast membranes equivalent to 0.4 µg chlorophyll (100%) from the wild type and *pgr7*, along with a series of dilutions of the wild-type sample.

To investigate the mechanism by which the *pgr7* mutation partially impairs electron transport between Q_A_ and P700, the accumulation of major photosynthetic complexes was compared between *pgr7* and the wild type. Immunoblot analysis using specific antibodies raised against the D1 subunit of the PSII reaction center, the PsaA subunit of the PSI reaction center, cytochrome *f* (cytochrome *b*_6_ *f* complex) and RbcL (large subunit of Ribulose 1,5-bisphosphate carboxylase/oxygenase) showed that all the complexes accumulated in *pgr7* as in the wild type (Figure 5). We conclude that PGR7 is not essential for the accumulation of a major photosynthetic complex. Plastocyanin, the soluble electron carrier between the cytochrome *b*_6_ *f* complex and PSI, also accumulated to the wild-type level in *pgr7* (Figure 5).

### Positional cloning of *PGR7*

To determine the genetic basis for the phenotypes of *pgr7*, we determined the segregation ratio of wild type to mutant in F_2_ plants of wild type x *pgr7*. Using digital video imaging, 115 progeny showed a wild-type phenotype, and 29 had a low NPQ phenotype among 144 F_2_ seedlings tested. In addition, the plant size and pigmentation phenotypes of *pgr7* cosegregated with the low NPQ phenotype (data not shown). A *G*-test determined that wild type to mutant phenotype ratio in F_2_ plants fitted to a 3∶1 ratio (χ^2^ = 1.90, P = 0.168) [15]. These results indicate that the mutant phenotypes of *pgr7* are caused by a recessive mutation in a single nuclear locus.

**Figure 6:**
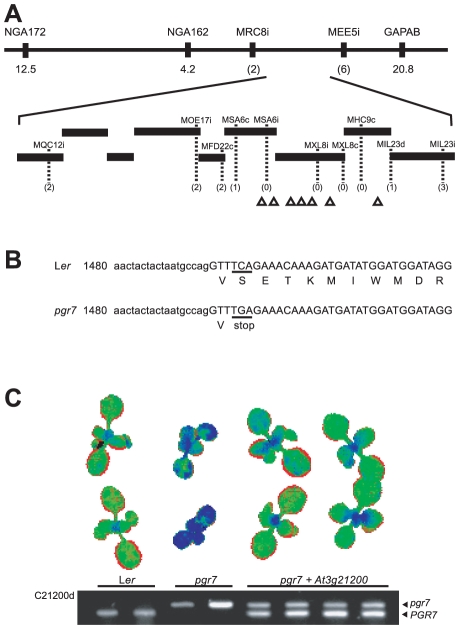
Cloning of the *PGR7* gene and complementation of *pgr7*. A) Positional cloning of *PGR7*. The horizontal line at the top represents chromosome 3 with marker names above and numbers below showing distances (in cM) from the corresponding marker to the *PGR7* gene. Numbers in parentheses are the number of recombinants detected. Bars represent BAC clones and the positions of genes encoding predicted chloroplast proteins are displayed with triangles. B) Partial DNA sequence of the *At3g21200* gene in *pgr7* and L*er*. The lower and upper cases indicate an intron and an exon region, respectively. The position of the transversion (C → G) in *pgr7* and the resulting stop codon are underlined. C) Complementation of the low NPQ phenotype of *pgr7* by the *At3g21200* gene. In the pseudo-color image, wild-type level of NPQ is displayed as green and low NPQ as blue. The agarose gel image shows genotype results determined by the dCAPS marker C21200d.

The affected gene in *pgr7* was identified by positional cloning. The mapping population plants were selected from the F_2_ plants resulting from crosses with a polymorphic wild-type strain (Col-0). Using simple sequence length polymorphism (SSLP) markers, we found that the *pgr7* mutation is located between NGA162 and GAPAB on chromosome 3 (Figure 6A). For fine mapping, we developed additional polymorphic markers based on the sequence polymorphisms published in the Monsanto Arabidopsis Polymorphism and L*er* Sequence Collection [16]. Genotyping results with these markers mapped the *pgr7* mutation between the MSA6c and MIL23d markers, which flank a region of genomic DNA defined by almost three bacterial artificial chromosome (BAC) clones (Figure 6A).

Within the region, many of the 45 annotated genes encode unknown proteins, and only a few of them were previously characterized or similar to known proteins. The phenotypes of the *pgr7* mutant strongly suggested that the *PGR7* gene might encode a chloroplast protein, so we identified seven candidate genes encoding predicted chloroplast proteins and determined their genomic DNA sequences in the *pgr7* mutant. The chloroplast localization was predicted by the ChloroP program [17]. Following sequence alignments of the wild type with the mutant, we found a C to G change in the *At3g21200* gene of *pgr7* (Figure 6B). The mutation occurred in an exon and resulted in a change of a TCA codon encoding serine (residue 234 of 317, including the putative chloroplast transit peptide) to a TGA stop codon in *pgr7*. This nonsense mutation would shorten the At3g21200 protein in the *pgr7* mutant by 84 amino acid residues at the C-terminal end (Figure 6B).

To confirm that the nonsense mutation in *At3g21200* is responsible for the low NPQ phenotype in *pgr7*, we performed a complementation experiment by introducing the wild-type *At3g21200* gene with its native upstream and downstream sequences into the *pgr7* mutant. We also developed a molecular marker, C21200d, to differentiate between the wild-type and mutant alleles. The results showed that all transformants contained both the mutant and the wild-type alleles of At3g21200, and the low NPQ phenotype of *pgr7* was returned to a wild-type level of NPQ (Figure 6C). In addition, the plant size of *pgr7* was restored to that of the wild type (Figure 6C). After transplantation into soil, the complemented plants were as green as L*er* (data not shown). Therefore, we were able to confirm that the mutation in *At3g21200* is responsible for the mutant phenotypes of *pgr7*.

### Chloroplast localization of PGR7

**Figure 7:**
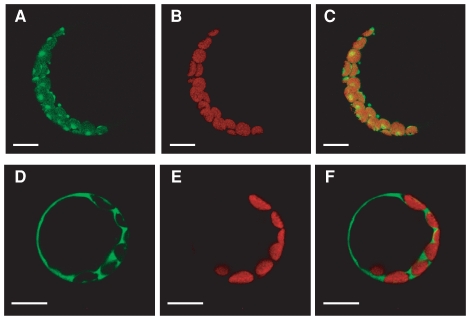
Chloroplast localization of a PGR7 N-terminus-GFP fusion protein in Arabidopsis leaf protoplasts. The protoplasts were transformed with either p21TPG3 encoding the PGR7 N-terminus-GFP fusion protein (A, B and C) or, as a control, p35Ω-SGFP(S65T) encoding GFP itself (D, E and F). The green fluorescence images (A and D) were overlaid with chlorophyll autofluorescence images (B and E) to generate merged ones (C and F). The bar represents 10 µm.

PGR7 was predicted to be a chloroplast protein, and it was detected by proteomics analysis of a chloroplast stroma fraction in Arabidopsis [18]. To confirm the chloroplast localization of PGR7, we examined the localization of a fusion with the green fluorescent protein (GFP). The N-terminal 41 amino acids were predicted to be a plastid-targeting signal by ChloroP. A DNA construct encoding an in-frame fusion of the first 115 amino acids of PGR7 with GFP was introduced into *Arabidopsis* leaf protoplasts, and then the subcellular localization of the green fluorescence was compared with that of chlorophyll autofluorescence using a confocal microscope (Figure 7). The green fluorescence emitted from the PGR7-GFP fusion protein colocalized with the chlorophyll fluorescence (Figures 7A–7C), showing that the fusion protein is targeted to chloroplasts. A very similar pattern was observed previously for another chloroplast protein, CpIscA [19]. In contrast, the green fluorescence from the GFP protein alone, without the PGR7 N-terminus, was observed only outside of chloroplasts (Figures 7D–7F). These results indicate that the PGR7 N-terminus contains a signal for chloroplast localization.

**Figure 8:**
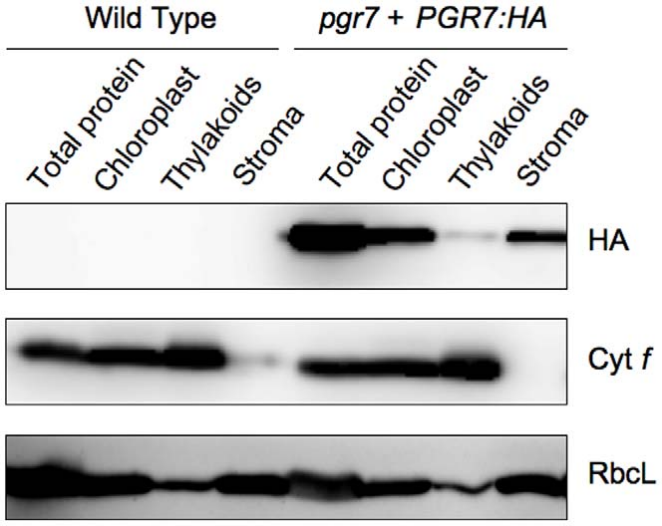
Chloroplast localization of an HA-tagged PGR7 protein. Immunoblot analysis of the HA epitope, cytochrome *f* (Cyt *f*), and RbcL was performed with proteins solubilized from leaves, chloroplasts, thylakoids, and stroma of the wild type and the *pgr7* mutant complemented with a *PGR7:HA* construct.

In addition, we complemented the *pgr7* mutation by expression of a full-length PGR7 protein with a hemagglutinin (HA) epitope tag at the C-terminus, under control of the 35S promoter. Immunoblot analysis of isolated chloroplasts and stromal and thylakoid membrane fractions revealed that the HA-tagged PGR7 protein is located in chloroplasts, predominantly in the stromal fraction (Figure 8), consistent with the available proteomics data [18]. A relatively small amount of HA-tagged PGR7 was also detected in thylakoids, similar to the distribution of the stromal protein RbcL but distinctly different from the thylakoid protein cytochrome *f* (Figure 8).

### Identification of conserved domains and homologs of PGR7

The *PGR7*/*At3g21200* gene model was confirmed by full-length cDNAs (GenBank accessions AY062654, AY087867 and BT002574). BLAST searches with the full-length protein sequence revealed the existence of homologs in vascular plants, the moss *Physcomitrella patens*, green algae (including *Micromonas sp.*, *Ostreococcus sp.*, and *Chlamydomonas reinhardtii*), diatoms, and the red alga *Cyanidioschyzon merolae*, as well as distant homologs in other eukaryotes, many bacteria, and archaea.

**Figure 9:**
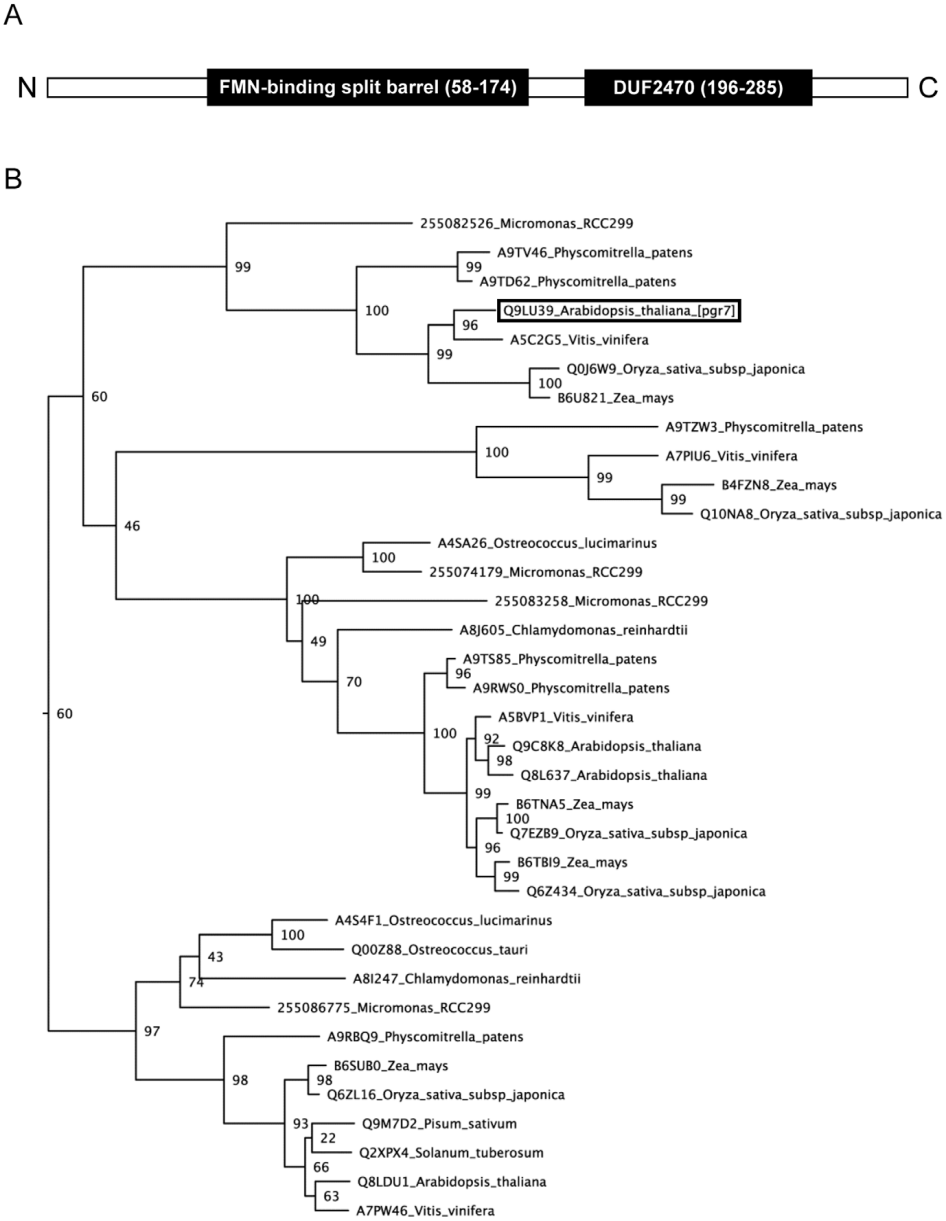
Identified domains and phylogenetic analysis of PGR7. A) Possible functional domains identified through Hidden Markov Model (HMM) construction and the positions of the FMN-binding split barrel domain and the DUF2470 domain in PGR7. B) Maximum likelihood phylogenetic analysis of PGR7 homologs in selected plants and green algae. A conserved region of PGR7 (residues 58-263) was used to identify 160 global homologs from plants, green algae, and bacteria in the UniProt database. The sequences were aligned, and a phylogenetic tree was generated with midpoint rooting. A branch of the larger tree (see Figure S1) that contains the plant and green algal sequences (with UniProt accession numbers) is shown here. The numbers at the nodes are bootstrap values from 100 runs.

Further bioinformatic analyses indicated that the mature PGR7 protein consists of two globular domains (Figure 9A), each having a distinct taxonomic distribution and three-dimensional structure. One domain, which spans residues 58 to 174, contains a predicted FMN-binding split barrel (IPR009002) (Figure 8A). This FMN-binding domain was previously detected in At3g03890 (UniProt accession Q8LDU1), which is one of three homologs of PGR7 that are encoded in the *Arabidopsis* genome. The second domain (residues 196 to 285) is a conserved domain of unknown function (PFAM DUF2470; IPR019595) that is also found in a family of cyanobacterial proteins. These cyanobacterial matches were also found by PSI-BLAST on the second iteration, and they appear to be single-domain proteins whose homology is restricted to the DUF2470 region of PGR7; thus, they lack the putative FMN-binding domain.

Because orthologs are most informative when they share a common overall domain architecture [20], we gathered homologs from the UniProt database and constructed a phylogenetic tree for an evolutionarily conserved region (residues 58 to 263) spanning the predicted FMN-binding split barrel domain and most of the DUF2470 domain (Figure S1). This analysis included 160 homologs with the same domain architecture from plants, green algae (but not other algae), and bacteria. A branch of the maximum likelihood tree containing PGR7 and 34 homologs from plants and green algae is shown in Figure 9B. Orthologs of PGR7 were evident in vascular plants, *Physcomitrella*, and *Micromonas*, whereas additional homologs were found in these species plus *Ostreococcus* and *Chlamydomonas*.

PSI-BLAST analysis also detected homology to several proteins with X-ray crystal structures in the Protein Data Bank (PDB). For example, PDB structure 2ARZ is a hypothetical protein from *Pseudomonas aeruginosa* detected by PSI-BLAST on the second iteration with an E-value of 7e-20 (15% identity and 34% positives). The region of homology detected by PSI-BLAST spans the length of PGR7 (excepting the predicted chloroplast transit peptide). Examination of the structure of 2ARZ shows two domains, a beta-barrel domain in the first half of the protein and a second domain that has a three-stranded antiparallel beta sheet and two helices.

## Discussion

The *pgr7* mutant was isolated in a video imaging screen for mutants that are defective in NPQ during illumination with high light, but the primary defect of the mutant appears to be in photosynthetic electron transport rather than in the NPQ machinery. Besides low NPQ, the plant size of *pgr7* was smaller than that of the wild type (Figure 2), and the chlorophyll content was also lower in *pgr7* compared to the wild type (Table 1). Similar phenotypes have been observed in mutants affected in various components of photosynthetic electron transport [21], [22], [23], [24], [25].

Like the *pgr1* [7], *pgr3* [26], *paa1* [8], *pgr5* [9], and *pgrl1* mutants [27], the NPQ defect of *pgr7* is attributable to a lower pH gradient across the thylakoid membrane due to restricted electron transport (Figure 3). For NPQ, acidification of the thylakoid lumen is necessary for activation of VDE in the xanthophyll cycle and for protonation of the PsbS protein [3]. The xanthophyll cycle pigments Z and A have both been shown to contribute to NPQ [28], and the de-epoxidation state [(A+Z)/(V+A+Z)] is highly correlated with NPQ [14]. The proportion of Z in the xanthophyll cycle pool [Z/(V+A+Z)] in *pgr7* was less than in L*er* under high light (Figures 4B and 4C), suggesting that VDE might be less activated in *pgr7* under high light, consistent with a defect in thylakoid lumen acidification due to restricted electron transport (Figure 3). However, the overall de-epoxidation state was similar between the mutant and the wild type (Figure 4A), and there was no significant difference in the xanthophyll cycle pool (V+A+Z) size (Table 1), suggesting that the slightly reduced xanthophyll cycle activity might not be sufficient to explain the NPQ defect of *pgr7*.

It is likely that incomplete protonation of PsbS is also contributing to the NPQ defect of *pgr7*. A complementation test performed by crossing *pgr7* with *npq4*, a mutant lacking PsbS [29], showed that the *PSBS* gene in the *pgr7* mutant is functional (Figure S2). However, protonation of PsbS as well as VDE is required for NPQ [5], [6]. When two glutamates, candidate amino acid residues for protonation in PsbS, were changed to glutamines, the mutated form was no longer able to complement the *npq4* mutation or bind N,N'-dicyclohexylcarbodiimide, an inhibitor of NPQ [5], [6]. A model for NPQ suggests that the protonation of PSII components including PsbS induces conformational changes required for NPQ [3]. Therefore, the restricted electron transport in *pgr7* may not be able to generate a high enough pH gradient to fully protonate PsbS, thus resulting in a lower level of NPQ.

In addition to linear electron transport, PSI cyclic electron transport significantly contributes to ΔpH formation during photosynthesis [9], [30]. The machinery of the PSI cyclic pathway(s) has not been clarified [31], and mutants defective in NPQ induction might provide clues to identify missing components. In *pgr5*, P700 is reduced at high light intensity due to the insufficient ATP supply for photosynthesis (Figure 3C). In contrast, P700 is more oxidized in *pgr7* compared to that in the wild type (Figure 3C). This phenotype is similar to that in the mutants partially defective in the intersystem electron transport such as *pgr1* [7], *pgr3* [26], and *paa1* [8]. It is possible that the restriction of electron transport between Q_A_ of PSII and P700 of PSI in *pgr7* affects both linear and PSI cyclic electron transport, but unlike *pgr5* [9] there does not seem to be a specific effect on PSI cyclic electron transport.

Besides low NPQ, the plant size of *pgr7* was smaller than that of the wild type (Figure 2), and the chlorophyll content was also lower in *pgr7* compared to the wild type (Table 1). Similar phenotypes have been observed in mutants affected in various components of photosynthetic electron transport [21], [22], [23], [24], [25]. The defect in electron transport between PSII and PSI in *pgr7* points to a possible impairment of the cytochrome *b_6_f* complex, however the accumulation of cytochrome *f* was not obviously affected (Figure 5 and Figure 8). A minor alteration in electron transport is unlikely to explain the growth defect and lower chlorophyll content of *pgr7*, because growth of the *pgr3* mutant, which has 50% lower accumulation of the cytochrome *b_6_f* complex, was indistinguishable from that of the wild type [26]. Future detailed analysis of cytochrome *b_6_f* activity, as well as oxygen evolution measurements, could help us to understand the phenotypes of *pgr7*. It is also possible that the *pgr7* mutation might have other, pleiotropic effects on chloroplast function. The presence of a putative FMN-binding split barrel domain in PGR7 (Figure 9A), along with the existence of PGR7 homologs in nonphotosynthetic bacteria (Figure S1), suggest an involvement of PGR7 in chloroplast redox biochemistry that might extend beyond the photosynthetic electron transport chain. Future work will aim to elucidate the specific biochemical function of PGR7 and its homologs.

## Materials and Methods

### Plant materials and growth conditions

The *pgr7* mutant was identified from fast neutron mutated M_2_ seeds (L*er*) obtained from Lehle Seeds (Round Rock, TX). Following 2∼3 days of stratification at 4°C, Arabidopsis plants were grown in a light (150 µmol photons m^−2^ s^−1^)- and temperature (22°C)-controlled growth chamber. Plants for chlorophyll fluorescence measurements were grown in a short-day condition (10 hours light/14 hours dark) for 5 to 6 weeks, and for other experiments, plants were grown in a long-day condition (16 hours light/8 hours dark).

### Digital video imaging and chlorophyll fluorescence measurements

Chlorophyll fluorescence images were acquired from 10∼14-day-old seedlings on MS-salt (Gibco, Grand Island, NY) agar plates using a video imaging system [13], [32]. The seedlings were grown in a light (80 µmol photons m^−2^ s^−1^)- and temperature (23°C)-controlled growth chamber. The video imaging system control and NPQ pseudo-color image generation were done by *IP lab* software (Scanalytics Inc., Fairfax, VA).

Chlorophyll fluorescence parameters were measured on attached rosette leaves using an FMS2 fluorometer (Hansatech, King's Lynn, UK) after overnight dark-adaptation. The maximum fluorescence after dark-adaptation (F_m_) and the maximum fluorescence in light-adapted condition (F_m_') were measured by applying a saturating pulse of light. F_o_ is the minimum fluorescence level in the dark-adapted condition. The maximum quantum yield of PSII (F_v_/F_m_) was calculated as (F_m_-F_o_)/F_m_, and NPQ was calculated as (F_m_-F_m_')/F_m_'. The NPQ measurements were conducted during actinic light illumination (1200 µmol photons m^−2^ s^−1^) for 10 min, followed by darkness for 5 min.

For measurement of the quantum yield of PSII (Φ_PSII_) and PSII reduction state (1-qL), plants were overnight dark-adapted and then exposed to 5 min illumination with a series of light intensities. Φ_PSII_ was calculated as (F_m_'-F_s_)/F_m_' with F_m_' measured by applying a saturating pulse of light at the end of each illumination and F_s_ being steady-state fluorescence during the illumination [33]. qL was calculated as F_o_' (F_m_'-F_s_)/F_s_(F_m_'-F_o_') [34] with F_o_' being minimum fluorescence after removal of the illumination. Statistical analyses and graph generation were performed using SPSS Graduate Pack v12 (SPSS Inc., Chicago, IL) and Microsoft Excel.

### Measurement of the Redox State of P700

Redox changes in P700 were assessed by monitoring the absorbance at 820 nm with a PAM chlorophyll fluorometer with an ED 800T emitter-detector unit as described [35]. The reduction state of P700 was calculated as 1-(ΔA_820_/ΔA_820_max). *In vivo* P700^+^ was recorded during actinic light illumination as ΔA_820_. The maximum *in vivo* content of P700^+^ (ΔA_820_max) was estimated by the absorbance change induced by far-red light illumination (720 nm, 0.66 µmol photons m^−2^ s^−1^).

### Pigment analyses

Leaf disks were treated in high light (1100 µmol photons m^−2^ s^−1^) on top of water for 5, 10, 30 and 60 min. Pigments were extracted and analyzed as previously described [36].

### Protein Analysis

Chloroplast isolation was performed as described previously [9]. Chloroplasts were burst by suspension in a medium of 20 mM HEPES/KOH (pH 7.6), 5 mM MgCl_2_, and 2.5 mM EDTA and centrifuged at 7,700×*g* for 3 min to separate the stromal fraction (supernatant) from the fraction containing the thylakoid membranes and chloroplast envelopes (precipitate). Proteins were separated by 10% SDS-PAGE, transferred to a polyvinylidene difluoride (PVDF) membrane, and detected as described previously [37].

### Molecular marker generation and positional cloning

Among F_2_ plants from *pgr7* x Col-0 crosses, low NPQ plants were selected using the digital video imaging system to generate a PGR7 mapping population. From individual plants of the mapping population, genomic DNA was extracted using an alkali method [38].

From the Arabidopsis Information Resource (TAIR), information on SSLP markers was downloaded; and from the Monsanto Arabidopsis Polymorphism and L*er* Sequence Collection [16], polymorphism information was obtained to generate polymorphic makers. Primers were designed using the web-based Primer3 program [39]. Markers were named as follows: BAC clone name where the polymorphisms locate and marker type abbreviation; i for insertion/deletion (IN/DEL) marker, c for cleaved amplified polymorphic sequences (CAPS) marker and d for derived CAPS (dCAPS) marker (Figure 6A).

Near NGA162, IN/DEL markers MRC8i (F: TGCGTTTTCACCTCCATACC; R: GGACAACCAAATAGAATGTTAGCC) and MQC12i (F: AAATCCTCCTCCAGCTCCAC; R: AGACATTCTTCCACCATCCAG) were generated based on 22 bp deletion and 18 bp insertion in Col. Close to the marker position of GAPAB, IN/DEL markers MEE5i (F: GACGAGAGCAGACTAACTTCAGG; R: TTGGAGGAAGAGAAAACAGAGG) and MIL23i (F: AGTTCGTCGTTAGGGTTTGG; R: AGAAACGTAATCTTGCGATGG) were designed based on 19 bp and 25 bp insertion, respectively, in Col-0.

On the same BAC clone as MIL23i, a dCAPS marker MIL23d was developed based on a mismatch selected in dCAPS Finder 2.0 [40]. In the forward primer (5′- AAGACTAGCGAAGAGTGaTT-3′), a mismatch nucleotide, the lower case a, was incorporated to generate a *Hin*f I restriction site when Col genomic DNA was used as a template in a PCR reaction with the reverse primer (5′-TTCGACACATGAAACTCACC-3′). After being digested with *Hin*f I, PCR products were resolved in 3% agarose gels.

Next to MIL23d, two CAPS markers MHC9c (F: CATCATGAGTATGTTGTGTGATAGTG; R: TCGTAGTGCTTTTGCTGTGG) and MXL8c (F: GGGTGATGTTACAATACACAAGAGG; R: ACGTGGGAAATCAGCATGG) and an IN/DEL marker MXL8i (F: TGTTTGTTTCCGGGGTAAAG; R: TGGATACGCCTGTCTTTGTG) were designed. The PCR products of MHC9c and MXL8c were digested with *Hin*f I and *Sau*3A I, respectively, and then polymorphisms of these three makers were resolved in 3% agarose gel.

From the other side of *pgr7*, IN/DEL marker MOE17i (F: TGATGGGTTTGGATCGATAG; R: GATGGCTATCACCAATATGAAATC) and MSA6i (F: CTCACACACTGAGCCATTCC; R: TGGAGGTGGTCTTAGGTTCC) were generated based on 15 bp and 54 bp insertions in Col-0. In addition, two CAPS markers MFD22c (F: GCGTGGTCACAAGTCAAAGC; CACATTCTTGATCGTCTTCTGC) and MSA6c (F: CGTGTTGAGTCTTGGTCAGC; R: CTCCGTTTGGTTTTATTCTTGG) were developed, and the PCR products of MFD22c and MSA6c were digested with *Hae* III and *Spe* I, respectively.

### C21200d marker and complementation

The C21200d marker was developed to confirm the mutation in *pgr7*. The dCAPS Finder v2.0 was used to find mismatches, and two mismatches (from GT to aa) were included in the forward primer (5′-CCAAAACTACTACTAATGCCAGaaTT-3′). In PCR with the reverse primer (5′-GGATGATTTTGCTCCCTTTTC-3′), PCR products amplified from wild-type genomic DNA contain an *Eco*R I recognition site (GAATTC), while those amplified from *pgr7* do not. The 22 bp polymorphism generated by *Eco*R I digestion was resolved in 3% agarose gels.

The 4.6 kb genomic DNA region encompassing the *PGR7* gene was amplified, for complementation, using *Pfu Turbo* DNA polymerase (Stratagene, La Jolla, CA) with the forward primer C200G2F (5′-CCCAAGCTTGTGCATCTCCCTGAGCTTTC-3′) and the reverse primer C200G2R (5′-CGCGGATCCGCGCCACTTTACCCAATAAG-3′). In the forward primer, a *Hin*d III recognition site and in the reverse primer, a *Bam*H I recognition site was added at the 5′-end of each primer. The PCR product was digested with *Hin*d III and *Bam*H I and then ligated into pBI121ΔGUS (kindly provided by Dr. Sheng Luan at the University of California, Berkeley) digested with *Hin*d III and *Bam*H I to generate p200CG2. The Agrobacterium-mediated floral dip method was used to introduce p200CG2 into the *pgr7* mutant [41]. T_1_ seeds were selected on MS-salt containing kanamycin (50 µg/ml), and then resistant plants were transplanted to determine NPQ levels.

A *PGR7:HA* construct encoding a version of the protein with a hemagglutinin (HA) tag at the C-terminus was amplified from a *PGR7* cDNA template using *Pfu Turbo* DNA polymerase (Stratagene, La Jolla, CA) with the forward primer 5′-CGCGGATCCGGGAATTCGATTAGCAACCA-3′ and the reverse primer 5′-CCGCTCGAGTCAAGCGTAATCTGGAACATCGTATGGGTATTGTCCTCCTCCCTTGTGA-3′). In the forward primer, a *Bam*H I recognition site and in the reverse primer, an *Xho* I recognition site was added at the 5′-end of each primer. The PCR product was digested with *Bam*H I and *Xho* I and then ligated into the binary vector pMD1 to generate p21HAK, for expression under control of the 35S promoter. Transformation into the *pgr7* mutant and screening of complemented lines was performed as described above.

### Localization of PGR7-GFP fusion protein

The Arabidopsis genomic DNA region encoding the first 115 amino acids of PGR7 was amplified with the forward primer 21TPG3F (5′-ACGCGTCGACAGCAACCAccATGCAACTCC-3′) and the reverse primer 21TPG3R (5′-GAACATccAtgGCAGACCTCTTGT-3′) using *Pfu Turbo* DNA polymerase (Stratagene, La Jolla, CA). In the forward primer, a *Sal* I recognition site and two mismatch nucleotides (from AA to cc) were added for cloning and higher translation efficiency, respectively. In the reverse primer, four mismatches, shown in lower case, were incorporated to generate an *Nco* I recognition site. The PCR product, following digestion with *Sal* I and *Nco* I, was ligated into the GFP reporter vector, p35Ω-SGFP(S65T) [42], to generate p21TPG3. Plasmid preparations were performed using the Qiafilter Plasmid Maxi kit (Qiagen, Valencia, CA).

Protoplast isolation and analysis of the subcellular location of transiently expressed GFP fusions by confocal fluorescence microscopy were performed as described previously [43].

### Bioinformatics analysis

NCBI annotation of the PGR7 amino acid sequence identified only the PFAM DUF2470 domain. Additional matches were inferred using tools in the PhyloFacts phylogenomic encyclopedia [44]. We used two main tools: HMM-based classification to PhyloFacts families to identify individual domains, and the PhyloBuilder web server [45] to construct phylogenetic trees for selected regions of PGR7. PhyloBuilder provides a pipeline for automated phylogenomic analysis and domain prediction, starting with gathering homologs using the FlowerPower software [46]. FlowerPower is similar to PSI-BLAST except that a set of subfamily hidden Markov models (HMMs) is used during the iterated search instead of a single profile during the iterated search. Default PhyloBuilder settings were used, with the exception of the number of subfamily HMM iterations in FlowerPower, which was set to 10 to increase the number of homologs retrieved. Homologs gathered using FlowerPower were aligned using MUSCLE [47], followed by alignment masking and phylogenetic tree construction, and identification of functional subfamilies using the SCI-PHY algorithm [48]. To identify homologous PFAM domains, we derived a consensus sequence from the multiple sequence alignment and scored it against PFAM HMMs using the hmmpfam software. We also identified homologous PDB structures using HMM scoring of PDB using an HMM constructed for the family using the UCSC SAM w0.5 software.

### Amino acid sequence alignment and phylogenetic analysis

A conserved region of PGR7 (residues 58-263) was submitted to the PhyloBuilder server (http://phylogenomics.berkeley.edu/phylobuilder) for homolog identification against the UniProt database. The resulting PhyloFacts book can be found with the accession number bpg081317. Detected homologs were retrieved, and the program uniqueseq (SAM v3.5) (http://compbio.soe.ucsc.edu/sam.html) was used to discard duplicate sequences, followed by manual verification. Four homologs from *Micromonas sp.* RCC299, detected by BLASTP search at NCBI, were added to the homolog list for a total of 160 sequences. MAFFT (http://align.bmr.kyushu-u.ac.jp/mafft/software/) was used to create a multiple sequence alignment, using the maxiterate option 100 times [49]. Masking was done in Belvu (http://sonnhammer.sbc.su.se/Belvu.html), by removing columns with more than 50% gaps. The alignment was submitted to the RAxML [50] web server hosted by the Cyberinfrastructure for Phylogenetic Research, CIPRES PORTAL v1.14 (http://www.phylo.org/) for maximum likelihood tree analysis with specified parameters: Substitution matrix  =  JTT; Maximum likelihood search; Random seed for bootstrapping  = 12345; Number of bootstrapping runs  = 100). The phylogenetic tree was rooted using the midpoint method (placing the root at the midpoint of the longest span).

## Supporting Information

Figure S1 Maximum likelihood phylogenetic tree of PGR7 homologs.
(1.04 MB TIF)

Figure S2 Results of complementation test between *pgr7* and *npq4*. The maximum fluorescence image after dark-adaptation (Fm) was captured after 30 min dark-adaptation; and then the maximum fluorescence image in the light-adapted condition (Fm') was captured after 2 min actinic illumination (500 µmol photons m-2 s-1).
(0.84 MB EPS)
