# Supplementary figures and images for: Arabidopsis thaliana PGR7 Encodes a Conserved Chloroplast Protein That Is Necessary for Efficient Photosynthetic Electron Transport
Source: PLoS One. 2010 Jul 21;5(7):e11688. doi: 10.1371/journal.pone.0011688 (PMC2908147; doi:10.1371/journal.pone.0011688)

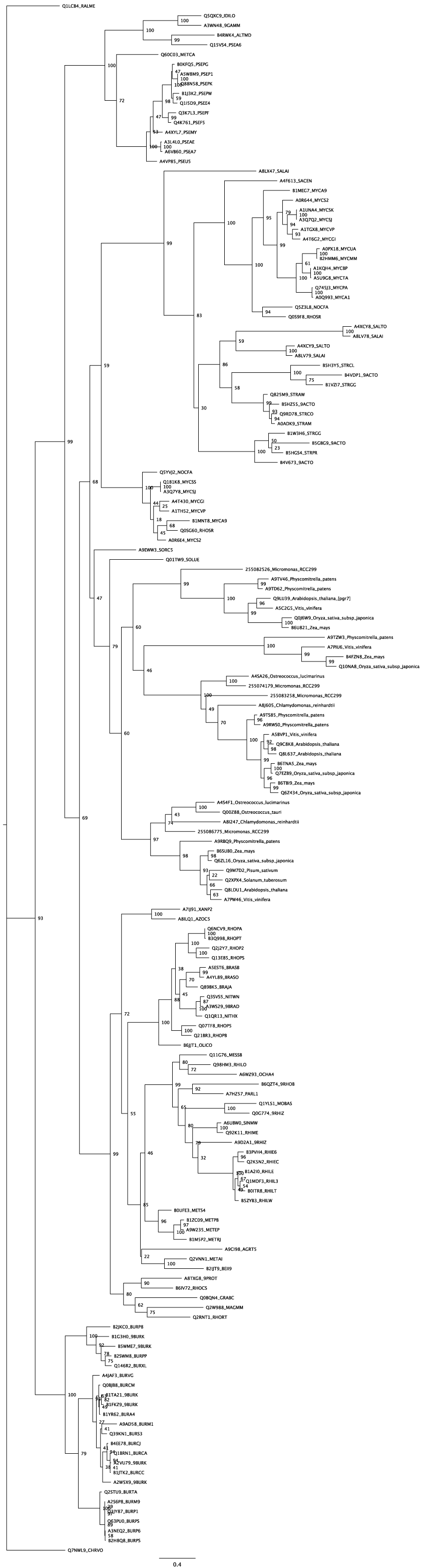

Supplement: Figure S1 — Maximum likelihood phylogenetic tree of PGR7 homologs. (1.04 MB TIF) [file pone.0011688.s001.tif]
